# Amelioration of intracellular stress and reduction of neural tube defects in embryos of diabetic mice by phytochemical quercetin

**DOI:** 10.1038/srep21491

**Published:** 2016-02-18

**Authors:** Lixue Cao, Chengyu Tan, Fantong Meng, Peiyan Liu, E. Albert Reece, Zhiyong Zhao

**Affiliations:** 1 Department of Obstetrics, Gynecology and Reproductive Sciences, University of Maryland School of Medicine, Baltimore, Maryland, USA; 2 Department of Biochemistry and Molecular Biology, University of Maryland School of Medicine, Baltimore, Maryland, USA; 3 College of Marine Technology and Environment, Dalian Ocean University, Dalian, China

## Abstract

Diabetes mellitus in early pregnancy causes birth defects, including neural tube defects (NTDs). Hyperglycemia increases production of nitric oxide (NO) through NO synthase 2 (Nos2) and reactive oxygen species (ROS), generating nitrosative and oxidative stress conditions in the embryo. The present study aimed to target nitrosative stress using a naturally occurring Nos2 inhibitor, quercetin, to prevent NTDs in the embryos of diabetic mice. Daily administration of quercetin to diabetic pregnant mice during the hyperglycemia-susceptible period of organogenesis significantly reduced NTDs and cell apoptosis in the embryos, compared with those of vehicle-treated diabetic pregnant mice. Using HPLC-coupled ESI-MS/MS, quercetin metabolites, including methylated and sulfonylated derivatives, were detected in the conceptuses. The methylated metabolite, 3-O-methylquercetin, was shown to reduce ROS level in embryonic stem cells cultured in high glucose. Quercetin treatment decreased the levels of Nos2 expression, protein nitrosylation, and protein nitration, alleviating nitrosative stress. Quercetin increased the expression of superoxide dismutase 1 and 2, and reduced the levels of oxidative stress markers. Expression of genes of redox regulating enzymes and DNA damage repair factors was upregulated. Our study demonstrates that quercetin ameliorates intracellular stresses, regulates gene expression, and reduces embryonic malformations in diabetic pregnancy.

Diabetes mellitus in early pregnancy increases the risk of birth defects in infants, a complication known as diabetic embryopathy^1^,^2^. Structural abnormalities, including exencephaly and spina bifida, most commonly occur in the central nervous system as the result of incomplete closure of the neural tube during early embryogenesis^3^,^4^. Collectively, these anomalies are referred to as neural tube defects (NTDs), and cause significant morbidity and mortality^5^,^6^.

Manifestation of NTDs is associated with excessive programmed cell death (apoptosis) in the neural folds, which, under hyperglycemia-induced intracellular stress conditions, is executed by pro-apoptotic factors such as caspase-8 and caspase-3^7^,^8^.

In diabetic pregnancy, maternal hyperglycemia elevates levels of nitric oxide (NO) in the embryo, by upregulating NO synthase 2 (Nos2), also known as inducible Nos (iNos)^9^,^10^,^11^. High levels of NO and associated reactive nitrogen species (RNS), such as peroxynitrite, augment protein nitrosylation at cysteine (S-nitrosylation) and nitration at tyrosine (nitrotyrosine) residues^12^,^13^. The global changes in these protein modifications lead to abnormal functions of proteins and organelles, a condition known as nitrosative stress^11^.

Maternal hyperglycemia also alters the morphology and function of mitochondria and elevates levels of reactive oxygen species (ROS) in the embryo^14^,^15^. In addition, it diminishes endogenous antioxidative buffering, including depletion of antioxidants, such as glutathione, and reduction of the expression and activity of antioxidative enzymes, including superoxide dismutases (SODs) and glutathione peroxidases (GPXes), resulting in oxidative stress^16^,^17^.

NO and RNS affect respiration of mitochondria, leading to over-generation of ROS^18^,^19^. They can also attenuate cell’s antioxidative capacity, for example, via nitration of SOD1 under hyperglycemic conditions^20^. In diabetic pregnant mice, inhibition of Nos2 is associated with decreases in protein oxidation^21^.

Strategies that aim to decrease maternal diabetes-induced oxidative stress and prevent embryonic malformations in diabetic animals have been explored^1^,^3^,^22^. However, using antioxidants that scavenge ROS, such as vitamins C and E, have not been appealing therapies to prevent birth defects because these agents have failed to ameliorate oxidative stress in other human diseases^23^,^24^.

Nitrosative stress is another target for intervention to prevent birth defects. Studies have shown that embryos without the *nos2* gene have lower malformation rates even when exposed to maternal diabetes^9^. In addition, oral treatments of diabetic pregnant mice with Nos2-specific inhibitors decrease abnormalities in offspring^9^,^21^. Therefore, strategies to target nitrosative stress, which may lead to alleviation of oxidative stress and produce long-lasting effects, warrant further exploration.

Strategies that will prevent birth defects in humans should not only be easily administered, such as through dietary supplementation, but also safe for pregnant women and their developing babies. Therefore, naturally occurring phytochemicals that can ameliorate nitrosative and/or oxidative stress have garnered attention as candidate agents because they can potentially overcome these challenges.

Quercetin is an aglycone form of flavonol, which is abundant in fruits and vegetables. It is a three-ringed organic phytochemical with attached hydroxyl groups, which can be metabolized and play a pivotal role in quercetin’s therapeutic effects^25^. Quercetin has been shown to inhibit the NOS2 enzyme in inflammation^26^,^27^,^28^, and, as a dietary supplement, can ameliorate disease conditions, including cardiovascular diseases, hypertension, and metabolic syndrome, without evident side effects^29^. In addition, animal studies have shown that quercertin can protect embryos from toxic hydroxyurea^30^. All these data implicate quercetin as a promising candidate to protect embryos from hyperglycemic insult in diabetic pregnancies. Therefore, we investigated whether quercetin ameliorate cellular stress and reduce embryonic abnormalities in diabetic embryopathy.

## Results

### Effect of quercetin on reduction in NTDs

**Figure 1:**
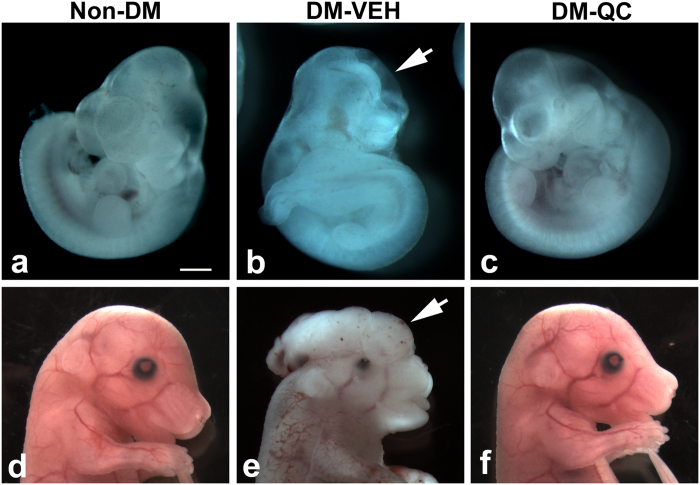
Effect of quercetin on NTD formation in diabetic pregnancies. (**a–c**) E10.5. (**d–f**) E15.5. (**a,d**) Non-DM. (**b,e**) DM-VEH. (**c,f**) DM-QC. Arrows in b and e indicate open neural tube and exencephaly, respectively. Scale bar = 5 mm in (**a–c**); 8 mm in (**d–f**).

After treatments with quercetin (QC), we examined the effects on NTD formation in the embryos of diabetic (DM) mice. In the diabetic mice treated with vehicle (VEH; DM-VEH group), the embryos had an open forebrain, midbrain, hindbrain, or forebrain and midbrain. Treatment with QC (DM-QC group) significantly decreased the NTD rate (1.4%), compared with that (23%) in the DM-VEH group (Table 1; Fig. 1), and similar to that in the group of non-diabetic controls (Non-DM; Table 1).

### Decreases in cell apoptosis in the neural tube by quercetin treatment

**Figure 2:**
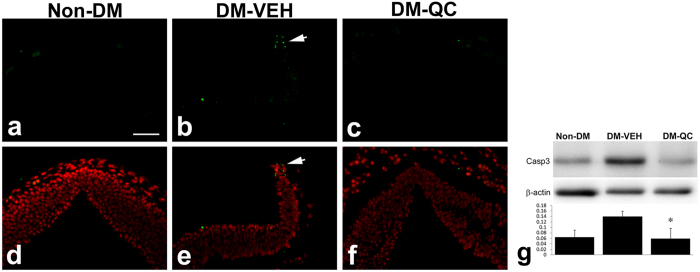
Apoptosis in the neural tissue of the embryos of diabetic pregnancies. (**a–c**) TUNEL assay of E10.5 embryos in the posterior midbrain. Positive signals are green. (**d–f**) Merged images of TUNEL and DAPI (red) staining. (**a,d**) Non-DM. (**b,e**) DM-VEH. Arrow indicates TUNEL positive cells in the dorsal region of the neural tube. (**c,f**) DM-QC. Scale bar = 50 μm in all images. (**g**) Western blot assay of cleaved caspase-3. Upper pane: Western blots; lower panel: quantification of blot band density (mean ± SD); *p < 0.05 (DM-VEH vs. Non-DM; DM-VEH vs. DM-QC; n = 4).

Apoptosis has been shown to be associated with NTDs in diabetic embryopathy^1^. We aimed to address the question of whether quercetin treatment affects apoptosis. In the DM-VEH group at E10.5, high levels of TUNEL (terminal deoxynucleotidyl transferase-mediated dUTP nick end labeling)-positive signals (apoptosis) were observed in the dorsal region of the neural tissues in the sites of NTDs (Fig. 2b,e). In the DM-QC group, fewer TUNEL-positive apoptotic bodies were seen in the dorsal neural tube of the embryos (Fig. 2c,f), in the similar level as in the Non-DM control group (Fig. 2a,d).

The decreases in apoptosis in the neural tube were confirmed using cleaved Caspase-3 as a marker. Levels of Caspase-3 were higher in the DM-VEH group than in the Non-DM group. In the DM-QC group, the levels of Caspase-3 were decreased significantly, compared with those in the DM-VEH group (Fig. 2g).

### Quercetin metabolites in the conceptus

**Figure 3:**
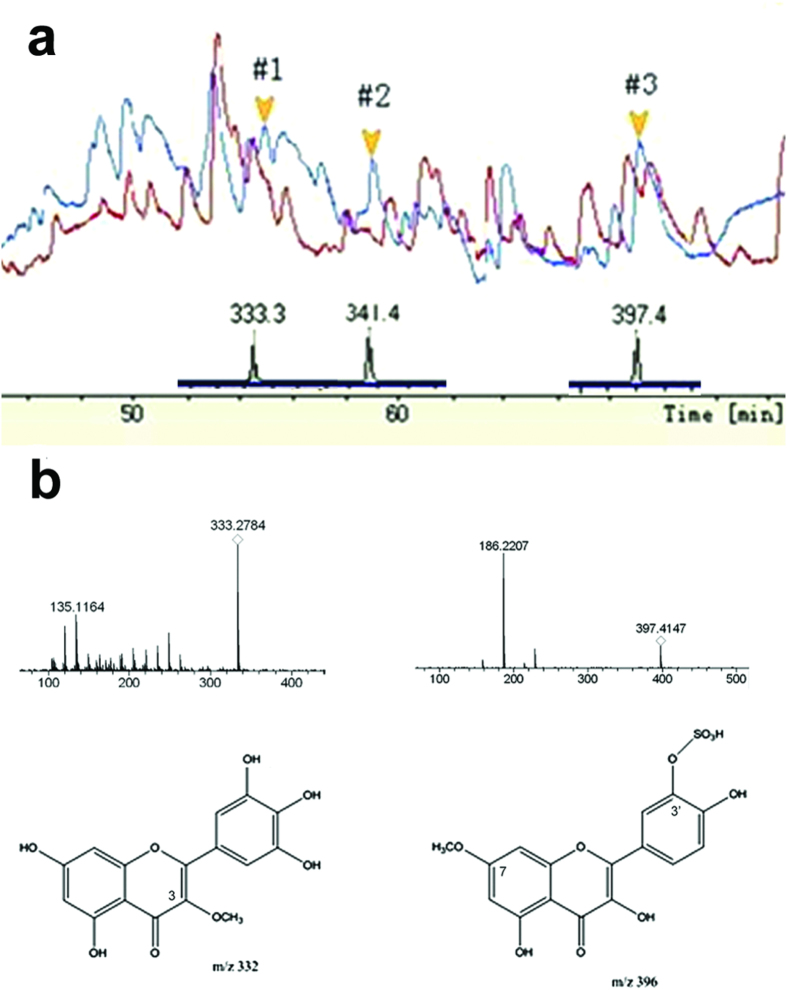
HPLC and ESI-MS/MS analyses of quercetin metabolites in E10.5 conceptuses. (**a**) HPLC (upper peaks) and in-source CID MS (lower peaks) assays. (**b**) High power MS post-source CID of peaks #1 and #3 and deduced quercetin metabolites.

To determine if quercetin underwent metabolism, passed through the maternal-embryonic interface, and reached the embryo, we used HPLC and ESI-MS/MS to detect quercetin metabolites in the E10.5 conceptuses. HPLC detected absorbance peaks (#1, #2, and #3) in the conceptuses of DM-QC group, which were not present in those of the DM-VEH group (Fig. 3a).

ESI-MS in-source CID revealed that these peaks contained molecules with m/z of 333.3, 341.4, and 397.4, respectively. MS spectrum analysis and database comparison showed that compounds #1 and #3 were likely quercetin metabolites. Compound #1 was a product of quercetin modification via replacing a hydroxyl group with a methyl group at the C3, C3′, or C4′ position (Fig. 3a). Compound #3 was potentially modified with a methyl group and sulfonic acid ester at either the C7 or C3′ position (Fig. 3a). High-power MS post-source CID generated fragments that were consistent with the frequent break-ups in quercetin metabolites (Fig. 3b)^31^,^32^, confirming that those compounds were quercetin metabolites.

### Effect of 3-O-methylquercetin on cellular homeostasis

**Figure 4:**
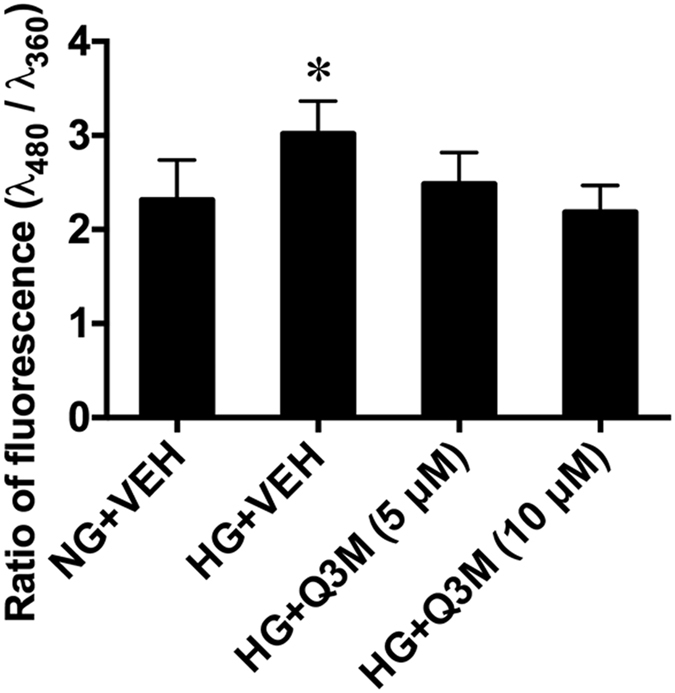
Effect of Q3M on ROS production in neural stem cells in high glucose. Quantification of fluorescence of an ROS dye (H_2_DCFDA) in neural stem cells (NE-4C) treated with Q3M in high glucose for 24 hours. *p < 0.05, comparison between HG +VEH and each of the other groups; n = 6 (repeated three times).

The MS assays showed that, after quercetin treatment, its metabolites reached the embryos. The question whether these quercetin derivatives exerted effects on embryonic cells remained to be addressed. We tested the effect of 3-O-methylquercetin (Q3M) on ROS generation in mouse embryonic neural stem cells. In the embryonic neural stem cells that were cultured in high glucose (HG; 33 mM) for 24 hours, levels of ROS were significantly increased, compared with those in the cells cultured in normal glucose (NG; 6 mM) (Fig. 4). Pilot experiments showed that Q3M reduced ROS levels in the cells exposed to high glucose in a concentration-dependent manner (2, 5, 10, and 20 μM), in comparison with the vehicle-treated HG group (HG-VEH). Further experiments determined that Q3M at 5 and 10 μM significant decreased ROS level (Fig. 4; n = 6 in triplicates), without affecting cell viability (data not shown).

### Effects of quercetin on nitrosative stress

**Figure 5:**
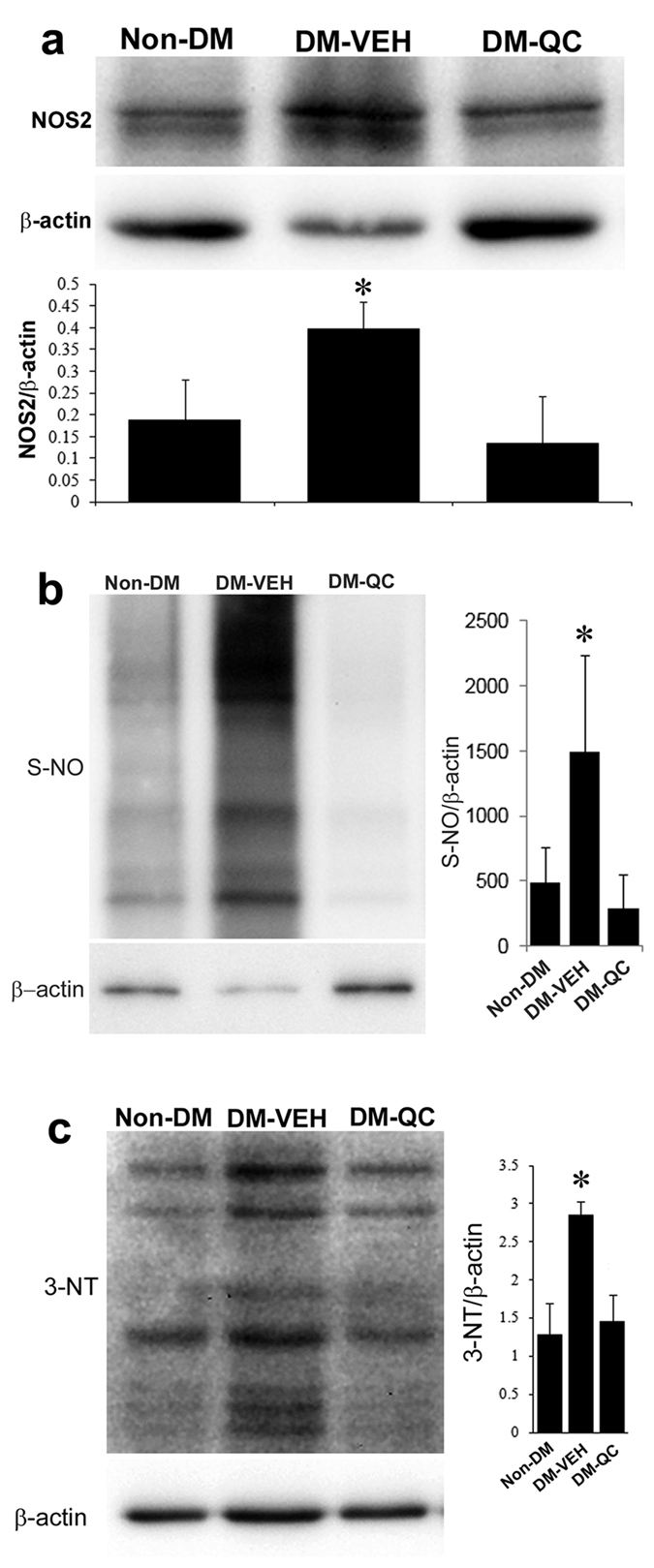
Alleviation of nitrosative stress by quercetin. Western blot assessments of protein expression in the neural tissues of E10.5 embryos. (**a**) Nos2. (Upper panel) Western blots; (Lower panel) Quantification of blot band density (Mean ± SD). (**b**) S-NO. (Left panel) Western blots; (Right panel) Quantification of blot band density (Mean ± SD). (**c**) 3-NT. (Left panel) Western blots; (Right panel) Quantification of blot band density (Mean ± SD). β-actin, loading control. * p < 0.05 (DM-VEH vs. Non-DM; DM-VEH vs. DM-QC; n = 4).

Quercetin has been shown to suppress the expression of Nos enzymes^26^,^27^,^28^. In the present study, we examined the expression of Nos1, 2, and 3 in the neural tissues of the embryos of diabetic mice, using immunoblotting assay. Nos2 expression was significantly increased in the DM-VEH group, compared with Non-DM group. Quercetin treatment significantly decreased Nos2 level (Fig. 5a).

Nitrosative stress is manifested by the increases in protein S-nitrosylation and nitration^11^. The levels of global protein S-nitrosylation in the neural tube of E10.5 embryos were significantly elevated in the DM-VEH group, compared with those in the Non-DM group (Fig. 5b). In the DM-QC group, the levels of protein S-nitrosylation were significantly lower than those in the DM-VEH group, but similar to those in the Non-DM group (Fig. 5b).

Protein nitration, indicated by 3-nitrotyrosine (3-NT), was significantly augmented in the DM-VEH group, compared with the Non-DM group (Fig. 5c). The levels of 3-NT were significantly reduced in the DM-QC group, compared with those in the DM-VEH group (Fig. 5c).

### Effects of quercetin on oxidative stress

**Figure 6:**
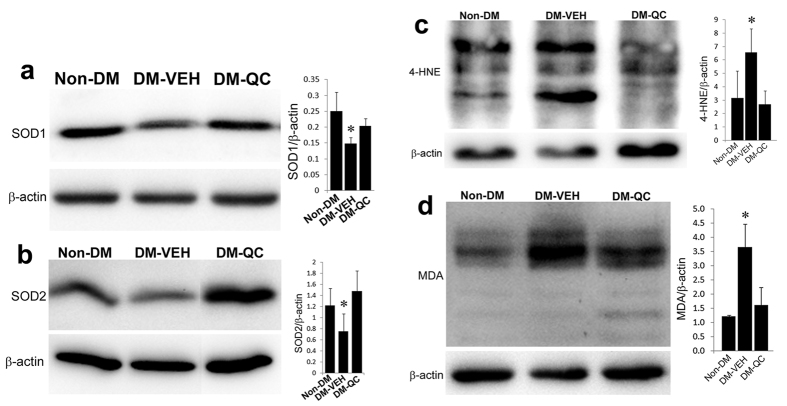
Alleviation of Oxidative stress by quercetin. Western blot assessments of protein expression in the neural tissues of E10.5 embryos. (**a**) Sod1. (Left panel) Western blots; (Right panel) Quantification of blot band density (Mean ± SD). (**b**) Sod2. (Left panel) Western blots; (Right panel) Quantification of blot band density (Mean ± SD). (**c**) 4-HNE. (Left panel) Western blots; (Right panel) Quantification of blot band density (Mean ± SD). (**d**) MDA. (Left panel) Western blots; (Right panel) Quantification of blot band density (Mean ± SD). β-actin, loading control. *p < 0.05 [(DM-VEH vs. Non-DM; DM-VEH vs. DM-QC; n = 4 (**a,b**); n = 3 (**c,d**)].

Sod1 and Sod2 are important antioxidative enzymes in regulating intracellular redox homeostasis and involved in diabetic embryopathy^1^,^2^. To investigate the effects of quercetin on Sod expression, we quantified their protein levels using immunoblotting. The levels of Sod1 and Sod2 in the DM-VEH group were significantly lower than in the Non-DM group (Fig. 6a,b). Quercetin treatment significantly increased the expression of these two proteins to the similar levels as in the Non-DM group (Fig. 6a,b).

By increasing Sod expression, quercetin could alter the redox state of the neural cells of the embryos. To address this question, we assessed oxidative stress markers in the neural tissues of the embryos. Levels of 4-hydroxynonenal (4-HNE) and malondialdehyde (MDA) were significantly increased in the DM-VEH group, compared with those in the Non-DM group, but significantly decreased in the DM-QC group, compared with those in the DM-VEH group (Fig. 6c,d).

### mRNA expression of stress-response genes in quercetin treatment

It has been suggested that quercetin affects gene expression at various levels^25^. Its effect on the expression of stress-response genes at the mRNA level was examined in the embryonic neural tissues using real-time PCR. The expression of a number of redox regulating and DNA damage response genes was significantly increased in the DM-QC group, compared with that in the DM-VEH group (Table 2). The antioxidative enzymes include mitochondrial Sod2 and Idh1 and cytosolic Prdx6. The DNA damage response factors include Ercc2, Hmgb1, and Nudt15 (Table 2).

## Discussion

Diabetes mellitus in early pregnancy increases the risk of congenital birth defects in infants^2^. Efforts have been made to explore strategies to prevent embryonic abnormalities^1^. The present study shows that phytochemical quercetin significantly decreases the rate of NTDs in the embryos of diabetic mice. Such effect is associated with significant decreases in cell apoptosis, alleviation of intracellular stress conditions, and expression of important factors in the neural tissues.

The present study also shows that quercetin is metabolized in the maternal system. Its derivatives pass through the maternal/embryonic interface to reach the embryos. At least, one metabolite, Q3M, exerts an effect on cellular homeostasis. All these suggest that orally administered quercetin, through its metabolites, may directly affect embryonic development.

Increased apoptosis in diabetic embryopathy is believed to be induced by intracellular signaling systems that are activated under aberrant conditions, including oxidative stress^1^,^33^. Alleviation of oxidative stress has been the main focus of research aimed at reducing developmental malformations^1^,^3^. However, approaches that utilize ROS scavengers are not preferred because clinical trials of antioxidant vitamins to alleviate oxidative stress have not had positive outcomes^23^,^24^. Therefore, strategies to deliver effective therapies to target other stress conditions warrant exploration.

Maternal hyperglycemia increases the production of NO, which itself, acts as a reactive nitrogen species (RNS), and activates an array of cell signaling pathways. Overproduction of NO in embryos of diabetic pregnancies is ascribed to upregulation of Nos2 (not Nos1 and Nos3)^34^,^35^. Experiments using *nos2* gene knockout animals demonstrate that Nos2 plays a key role in mediating the adverse effect of hyperglycemia on embryonic malformation^9^. Furthermore, treatments with Nos2 specific inhibitors in diabetic pregnant mice significantly reduce embryonic and fetal abnormalities in diabetic animals^9^,^21^. All these data suggest that Nos2-induced nitrosative stress is an important condition in diabetic embryopathy and a potential target for intervention to prevent birth defects.

The present study demonstrates that treatment with quercetin significantly decreases protein S-nitrosylation and tyrosine nitration, thereby alleviating nitrosative stress. It also reduces ROS production and oxidative stress. These effects result in significant decreases in apoptosis and NTDs in the embryos of diabetic mice.

NO is synthesized by three NOS enzymes in cells. Each of them reacts to different stimulation, with NOS2 being most sensitive to environmental induction. Nos1 and Nos3 are expressed at very low levels in early embryos. Nos1 is mainly present in mature neuron in fetus and adult. Nos3 (both mRNA and protein) is expressed in the primitive endothelial cells peripheral to the neural epithelium in embryos^9^,^36^. Nos2 is expressed in the neural epithelium of embryos at the malformation vulnerable stages. In this study, quercetin treatment significantly reduced Nos2 protein level. As Nos2 is important in diabetic embryopathy^9^, it is suggested that quercetin alleviates nitrosative stress via suppressing Nos2.

Quercetin may also inhibit Nos activity and scavenge RNS^37^. This implies that, in diabetic embryopathy, quercetin may suppress Nos2 expression and inhibit Nos2 enzymatic activity. Future work aims to address the latter and underlying mechanisms.

In addition to amelioration of nitrosative stress, quercetin also alleviates oxidative stress. It has been suggested that quercetin scavenges ROS in other systems^38^. However, its direct ROS scavenging activity in embryos of diabetic animals remains to be addressed.

ROS scavengers, such as vitamin C and E, can blunt the action of the toxic radicals. However, to achieve sustained effects requires constant administration of the antioxidants. Antioxidative enzymes, on the other hand, are endogenous components of the cellular defense system^39^,^40^. Once produced by cells under stimulation, these enzymes can provide effective and long-lasting protection to cells.

The present study shows that quercetin increases the expression of a number of antioxidative enzymes in the embryos of diabetic mice, including Sod1, Sod2, Prdx6, and Idh1. Prdx6 is a cytosolic protein that plays a role in scavenging ROS and inhibiting apoptosis^41^,^42^. Sod2 and Idh1 are localized in the mitochondria, which have been shown to produce ROS in diabetic embryopathy^14^. Sod2 directly scavenges ROS, while Idh1 catalyzes the production of antioxidant NADPH^43^. Elevated levels of Sod2 have been shown to be associated with a strain of rat that is resistant to hyperglycemia-induced embryonic malformations^44^. Overexpression of SOD1 in transgenic mouse embryos reduces NTD rate in diabetic pregnancies^45^,^46^. Taken together, the upregulation of these three enzymes suggests that quercetin augments the embryo’s endogenous cellular defenses against oxidative insult in diabetic pregnancy.

High levels of ROS damage DNA, which, when not repaired, can lead to apoptosis^47^. DNA damage has been detected in embryos from diabetic dams^48^; however, DNA repair activities have not been characterized. In the present study, quercetin upregulated genes associated with DNA repair, including Nudt15, Ercc2, and Hmgb1. DNA damage repair constitutes a line of protection against hyperglycemic insult and a target for developing interventions.

The goal of targeting intracellular stress in diabetic embryopathy is to prevent embryonic malformations. However, a number of hurdles need to be overcome to reach this goal. Human and animal studies have demonstrated that the period of development most vulnerable to maternal hyperglycemic insult is early embryogenesis (before seven weeks of gestation in humans and before E11 in mice)^49^,^50^. Thus, any interventions to protect the embryos from diabetes-induced damage should be applied before conception and during the early period of pregnancy. A practical approach for intervention is using dietary supplements, which must be effective and safe to both pregnant women and their developing babies.

Quercetin is a naturally occurring polyphenol in fruits and vegetables, and widely available as a dietary supplement^25^,^51^. It has been used to ameliorate many disease conditions in humans. Studies in humans and animals have shown that quercetin has very low toxicity when used at high doses for a short period of time, and at lower doses when taken for chronic conditions^51^,^52^, implying that it may be a good candidate agent to prevent birth defects.

It has been shown that quercetin, once ingested, is quickly absorbed by the intestines into the circulation and distributed to other organs in the body^51^,^53^. However, it remained unclear whether quercetin passes through the maternal/fetal interface to reach the embryo. Using HPLC and ESI-MS/MS, the present study is among the first to show that quercetin administered to the dams is present in the embryos in metabolized forms, suggesting that quercetin likely exerts direct effects, through its metabolites, on the embryo.

Quercetin is an aglycone member of the flavonol family, consisting of a large number of quercetin glycoside derivatives^25^. It is believed that the hydroxyl groups on the quercetin molecule are the active sites for metabolic modifications in the gastrointestinal tract and blood circulation^53^,^54^. In the embryo, quercetin metabolites were modified with methyl groups and sulfonic acid esters, with the core rings intact. Modifications of quercetin at different sites with different groups may alter its biochemical properties (e.g., solubility in water or lipid and permeability through cell membrane) and biological activities (e.g., antinitrosative and antioxidative)^51^. In the present study, methylated quercetin, Q3M, was shown to exert protective effects (at least antioxidative effects) in embryonic neural stem cells. Future work is aimed at investigating molecular interactions between quercetin derivatives and stress-associated proteins, such as Nos2, as well as transcription factors that regulate the expression of the stress response proteins in embryonic cells.

In summary, protecting embryos from maternal hyperglycemia insult may require a strategy which directly targets key cellular stress condition(s) in the embryo, provides sustained protection during sensitive period of embryogenesis, and is safe for pregnant women and their embryos/fetuses. Quercetin, a naturally-occurring phytochemical, appears to meet these requirements. The present study provides information for future pre-clinical studies to determine the effectiveness, efficacy, and safety aspects of clinical application of quercetin.

## Methods

### Diabetic animal model

This study was carried out in strict accordance with the recommendations in the Guide for the Care and Use of Laboratory Animals of the National Institutes of Health. The protocol was approved by the Institutional Animal Care and Use Committee of University of Maryland, Baltimore.

Female mice (C57BL/6J) were injected intravenously with streptozotocin (Sigma-Aldrich, St. Louis, MO) in 0.1 M citrate buffer (pH 4.5; 65 mg/kg body weight) to eliminate insulin-producing β-cells in the pancreas^55^. Diabetes mellitus (DM) was defined as blood glucose levels reaching ≥14 mM (250 mg/dl). Female mice injected with citrate buffer were used as non-diabetes mellitus (Non-DM) controls. Euglycemia (~8 mM) was restored in the diabetic mice by subcutaneous implantation of insulin pellets (Linshin Canada, Scarborough, ON, Canada)^56^,^57^. The female mice were paired with normal male mice of the same strain in the afternoon. The presence of the vaginal plug the next morning was designated as embryonic (E) day 0.5. At E5.5, insulin implants were removed to make the female mice hyperglycemic again from E6.5 before neurulation begins^58^.

Quercetin (QC; CAS 117-39-5; Cayman Chemical, Ann Arbor, MI), suspended in water (vehicle = VEH; 0.1 ml), was administered via gavage feeding to diabetic pregnant mice at 100 mg/kg body weight, once a day from E7.5 to E10.5. Maternal blood glucose levels were monitored every day from E6.5 to E10.5. Three groups were included in this study, Non-DM, DM-VEH, and DM-QC. At E10.5 (late neurulation^58^) and E15.5 (close to term), embryos were collected for examination of NTDs. Neural tissues from E9.5 and E10.5 were collected for biochemical and molecular assays.

### TUNEL assay

The embryos at E10.5 were fixed in 4% paraformaldehyde in phosphate buffered saline (PBS, pH 7.4) at 4 °C for 24 hours, and embedded in paraffin wax through dehydration with ethanol and xylene. Tissue sections in 6-μm thickness were cut using a microtome. Tissue sections were dewaxed in xylene and rehydrated through a reverse ethanol concentration series to water.

Tissue sections were treated with proteinase K (20 μg/ml) for 15 min at room temperature, incubated with TUNEL reagents containing dUTP-fluorescein (Roche, Indianapolis, IN) for 2 hours at 37 °C, and counterstained with 4′,6-diamidino-2-phenylindole (DAPI). The sections were observed under a fluorescent microscope (Zeiss, Thornwood, NY). Microscopic images were captured using a CCD camera connected to an image analysis station and arranged using the Adobe Photoshop software program. At least four embryos in each group were used for the assay.

### High performance liquid chromatography and mass spectrometry

The conceptuses at E10.5 were collected 4 hours after the last quercetin treatment to extract small organic molecules. Briefly, the conceptuses (3/sample, 4 samples/group) were homogenized in methanol and centrifuged (5,000 × g for 15 min) at 4 °C. The supernatants were collected and concentrated via evaporation with nitrogen gas.

Samples were assayed using high performance liquid chromatography (HPLC)-coupled high resolution electrospray ionization tandem mass spectrometry (ESI-MS/MS). HPLC (Agilent 1290; Agilent, Santa Clara, CA) was performed with a separation column (2.1 × 50 mm I.D. 1.8 μm) packed with SB zorbax C18. The mobile phase consisted of water (A) and gradient-grade acetonitrile (B), and run at a flow-rate of 0.25 ml/min in a gradient profile: 0–5 min 5% B in A; 5–25 min 5–50% B in A, linear; 25–60 min 50–90% B in A, linear; 60–61 min 90–98% B in A, linear; 61–86 min 98% B in A; 86–87 min 98–5% linear. An UV detector and ESI interface were set up to simultaneously monitor UV signals between wave lengths of 190–600 nm.

Quadrupole Time-of-Flight MS (microTOF QII; Bruker, Germany) was performed in a positive ionization mode under ESI source of 4.5 kV at 180 °C. Data were collected within a range of 50 to 1500 mass-to-charge ratio (m/z). Additional collision-induced dissociation (CID) was performed with collision energy of 15 and 30 eV. Raw data were analyzed using associated software (Data Analysis 4.0.msi, Bruker Compass, Germany) and databases (Metabolite Tools 2.0 SR1, Bruker Compass, Daltonics).

### Cell-based ROS assay

Neural stem cells (NE-4C; American Type Culture Collection), derived from 9-day-old mouse embryos, were plated in 96-well plates (2 × 10^4^ cells/well) in Dulbecco’s Modified Eagle Medium (DMEM, Life Technologies) containing 10% fetal bovine serum and 6 mM glucose (normal glucose or NG). After 24 hours of culture, the cells were treated with NG, high glucose (HG, 33 mM), and 3-O-methylquercetin (Q3M; Sigma-Aldrich; in dimethylsulfoxide as a VEH; 1:1000 in media) in HG in the following groups: NG-VEH, HG-VEH, HG-Q3M. After 24 hours of treatment, the cells were loaded with fluorescent dyes, 2′,7′-dichlorodihydrofluorescein diacetate (H_2_DCFDA) and Hoechst 33342 (Life Technologies), for 10 minutes at 37 °C, to stain ROS and the cell nuclei, respectively. After washed twice with FluoroBite DMEM (Life Technologies), levels of fluorescence were measured using a Biotek Synergy II microplate reader at 480 nm and 360 nm, respectively. The ratio of the fluorescent levels was subject to analysis.

### Western blotting

The neural tissues in the brain region of the E10.5 embryos were isolated and individually collected using fine scissors under a stereo-microscope in cold PBS (pH 7.4). The tissues were homogenized in a lysis buffer [25 mM Tris-HCl (pH 7.6), 150 mM NaCl, 1% Nonidet P-40, 1% sodium deoxycholate, 0.1% sodium dodecyl sulfate (SDS)] containing protease and phosphatase inhibitors (Thermo Scientific, Rockford, IL). The tissue homogenates were centrifuged at 14,000 rpm for 15 min at 4 °C to obtain supernatants.

Samples containing 20–40 μg of protein from normal embryos in the Non-DM and DM-QC groups, and malformed embryos in the DM-VEH group were resolved in 10% polyacrylamide gel using electrophoresis in the presence of SDS, and blotted onto polyvinylidene fluoride membranes (Millipore, Billerica, MA). After blocking with 10% non-fat milk or bovine serum albumin, the membranes were incubated with primary antibodies for 16 hours at 4 °C. The primary antibodies were against Nos1, Nos2, Nos3, Sod1, and Sod2 (Cell Signaling Technology, Beverly, MA), 3-nitrotyrosine (3-NT), 4-hydroxynonenal (4-HNE), and malondialdehyde (MDA; Millipore, Temecula, CA). This was followed by incubation with horseradish peroxidase (HRP)-conjugated secondary antibodies (Santa Cruz Biotechnology, Santa Cruz, CA) for 1 hour at room temperature. Signals were detected using SuperSignal West Pico Chemiluminescent Substrate (Thermo Scientific). Images were captured and density of the bands was measured using UVP Bioimage system (UVP, Upland, CA).

The same membranes were stripped using Restore Western Blot Stripping Buffer (Thermo Scientific) and probed again with an antibody against β-actin (Abcam, Cambridge, MA) to control equal loading of protein samples. The values of β-actin band density were used to normalize those of the corresponding bands of interest.

### Protein S-nitrosylation assay

Protein S-nitrosylation in the neural tissues of E10.5 embryos was assessed using an S-nitrosylated Protein Detection Assay kit (Cayman Chemicals, Ann Arbor, MI), following the manufacturer’s instructions. Briefly, neural tissues of E10.5 embryos were dissected using fine scissors under a dissecting microscope. The tissues were homogenized in lysis buffer (Buffer A) containing Blocking Agent, centrifuged to remove debris, and precipitated with acetone. The precipitated protein samples were resuspended in Buffer B containing Reducing and Labeling agents (biotin), and incubated for one hour at room temperature. The labeled protein samples were precipitated with acetone and resuspended in a buffer for detection using Western blotting with avidin-HRP. Signals were captured and measured using UVP Bioimage system (UVP, Upland, CA).

### Real-time polymerase chain reaction

Neural tissues in the brain regions of E9.5 embryos were isolated in cold PBS and collected individually. Total RNA was extracted from the tissues using RNeasy kit (Qiagen, Valencia, CA), following the manufacturer’s instructions. Genomic DNA contamination was cleaned with DNase I. cDNA was synthesized from the RNA samples using the RT (reverse transcription) First Strand kit (Qiagen). Real-time polymerase chain reaction (PCR) of mouse NO signaling array (PAMM-062Z) was performed using the Real-Time PCR kit (Qiagen), following the manufacturer’s instructions, on an ABI StepOne thermal cycler. Data were analyzed using a Qiagen online program to identify genes with significant differences (p < 0.05) in expression levels between the DM-VEH and DM-QC groups.

### Statistical analyses

NTD rate was calculated as a percentage of the embryos, with NTDs in total number of embryos. Log binomial models for clustered data were applied to compare the NTD rates between groups, with calculated confidence intervals. Ratios of fluorescence intensity at two different wavelengths and ratios of band density of interest to that of β-actin on Western blots, presented as Mean ± standard deviation (SD), were analyzed using the Student t-test. A p-value of < 0.05 was considered statistically significant.

## Additional Information

**How to cite this article**: Cao, L. *et al.* Amelioration of intracellular stress and reduction of neural tube defects in embryos of diabetic mice by phytochemical quercetin. *Sci. Rep.* **6**, 21491; doi: 10.1038/srep21491 (2016).

## Figures and Tables

**Table 1:** Effects of quercetin treatment on reduction in NTD rate.

	Non-DM	DM-VEH	DM-QC
NTD/total embryos (Litter)	0/61 (8)	19/83 (11)	1/72 (10)*
Maternal glucose (E7.5-E10.5; mg/dl; Mean ± SD)	103.20 ± 24.11	318.85 ± 94.74	290.09 ± 69.80

*p = 0.003, DM-VEH vs. DM-QC (95% CI 0.009, 0.3758). DM, diabetes; QC, quercetin; NTD, neural tube defect; VEH, vehicle.

**Table 2:** Real-time PCR assay of gene expression.

Gene Symbol	Description	Refseq	Fold Regulation	P value
Als2	Amyotrophic lateral sclerosis 2 homolog	NM_028717	1.29	0.005
Cd151	CD151 antigen	NM_009842	1.41	0.040
Dynll1	Dynein light chain LC8-type 1	NM_019682	1.32	0.026
Ercc2	DNA excision repair factor	NM_007949	1.67	0.019
Hmgb1	High mobility group box 1	NM_010439	1.86	0.004
Idh1	Isocitrate dehydrogenase 1 (NADP+)	NM_010497	1.54	0.010
Nudt15	Nucleoside diphosphate linked moiety X-type motif 15	NM_172527	1.33	0.047
Ppp1r15b	Protein phosphatase 1, regulatory subunit 15b	NM_133819	1.36	0.037
Prdx6	Peroxiredoxin 6	NM_007453	1.39	0.014
Scd2	Stearoyl-Coenzyme A desaturase 2	NM_009128	2.23	0.015
Sod2	Superoxide dismutase 2	NM_013671	1.28	0.005
Rb1	Retinoblastoma 1	NM_009029	−1.56	0.036

Comparison between DM-QC and DM-VEH. N = 4. Minus (−) indicates a decrease in expression.
